# Impact of Tohoku-Oki 3.11 M9.0 Earthquake on the Fault Slip Potential of the Active Quaternary Faults in Beijing City: New Insights from In Situ Stress Monitoring Data

**DOI:** 10.3390/s22134888

**Published:** 2022-06-29

**Authors:** Yulu Fan, Chengjun Feng, Peng Zhang, Bangshen Qi, Jing Meng, Chengxuan Tan

**Affiliations:** 1 Institute of Geomechanics, Chinese Academy of Geological Sciences, Beijing 100081, China; fanfyl@cags.ac.cn (Y.F.); zhangpeng@mail.cgs.gov.cn (P.Z.); qibangshen@mail.cgs.gov.cn (B.Q.); mengjing@mail.cgs.gov.cn (J.M.); tanchengxuan@mail.cgs.gov.cn (C.T.); 2 Key Laboratory of Active Tectonics and Geological Safety, Ministry of Natural Resources, Beijing 100081, China

**Keywords:** Beijing Plain area, Tohoku-Oki 3.11 M9.0 earthquake, in situ stress field, in situ stress monitoring, fault slip potential, Mohr–Coulomb failure criteria, Byerlee’s law

## Abstract

In order to ascertain the impact of the Tohoku-Oki 3.11 M9.0 earthquake on the stability of the faults in the Beijing Plain, we investigated the adjustment of the in situ stress field of the Beijing Plain after this earthquake based on in situ stress monitoring data. Then, we analyzed the stability of the five main faults in each adjustment stage of the in situ stress field based on the Mohr–Coulomb failure criteria and Byerlee’s law. Finally, we studied the fault slip potential (FSP) of the main faults under the current in situ stress field. The research results show that (1) after the Tohoku-Oki 3.11 M9.0 earthquake, the tectonic environment of the Beijing Plain area changed rapidly from nearly EW extrusion to nearly EW extension, and this state was maintained until June 2012. After this, it began to gradually adjust to the state present before the earthquake. As of September 2019, the tectonic environment has not recovered to the state present before the earthquake. (2) The ratios of shear stress to normal stress on the fault plane of the fault subsections in the three time periods before the Tohoku-Oki 3.11 M9.0 earthquake, 6 June 2012 and 8 September 2019 were 0.1–0.34, 0.28–0.52, and 0.06–0.29, respectively. It shows that the stress accumulation level of faults in the Beijing Plain area increased in a short time after the earthquake and then gradually decreased. (3) Under the current in situ stress field, most of the subsections of the five main faults have a low FSP (<5%). The areas with high FSP are mainly concentrated in the central and southeastern parts of the Beijing Plain, including the Nankou-Sunhe fault, the northern section of the Xiadian fault, and the areas where the five faults intersect.

## 1. Introduction

With the rapid increase in the urban population and the more frequent inter-city population mobility, urban agglomerations centered on big cities have become the main form of urbanization [1], and concern regarding the geological security of urban areas has become more prominent [2]. The Beijing Plain is the seat of the Chinese capital and the core of the Beijing-Tianjin-Hebei urban agglomeration. It is located at the intersection of the Taihang Mountain fault zone and the Zhangjiakou-Bohai fault zone. The stability of concealed active faults is a geological safety issue that should be the focus of long-term attention.

**Figure 1:**
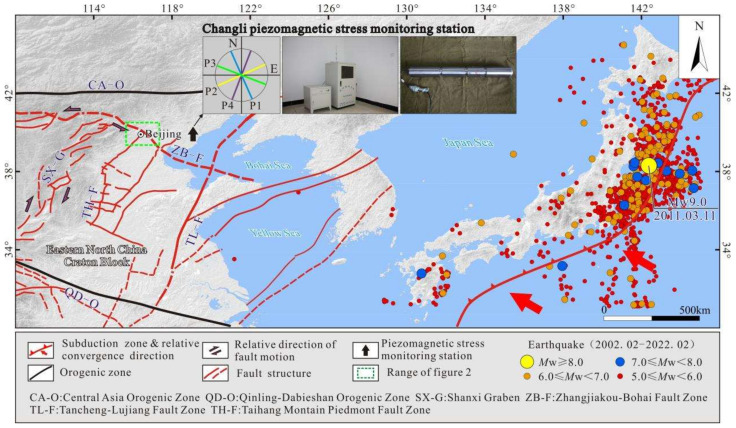
Regional structure of the Beijing Plain area and location of the Changli piezomagnetic stress monitoring station.

The Beijing Plain is located in the northern part of the North China Plain, and there are two main groups of NE-trending and NW-trending faults in the area (Figure 1). The NE-trending faults mainly include the Huangzhuang-Gaoliying fault, the Shunyi fault, the Nanyuan-Tongxian fault, and the Xiadian fault, and the NW-trending faults mainly include the Nankou-Sunhe fault. All of these faults have experienced significant activity since the Late Pleistocene [3,4,5], and they are the main earthquake-generating and seismic-controlling faults in the plain area. According to historical records and instrumental records: from 231 BC to the present, there have been 16 destructive *M*_S_ ≥ 5.0 earthquakes in the Beijing Plain area, of which the 1679 Sanhe-Pinggu *M*_S_8.0 earthquake had the largest magnitude [6]. From 1970 to the present, a total of 8026 earthquakes with magnitudes of 1.0–2.9, 58 earthquakes with magnitudes of 3.0–3.9, and 19 earthquakes with magnitudes of 4.0–4.9 have been recorded, but earthquakes with magnitudes of ≥5.0 have not been detected [7]. The epicenters of these earthquakes were concentrated in the areas where the main active faults intersect and along the active faults. Considering the fault structure, earthquake intensity, and frequency comprehensively, the Beijing Plain area has the tectonic background and conditions for the occurrence of moderate-intensity earthquakes in the future. In addition, the main active faults also have a controlling effect on geological disasters such as ground fissures and land subsidence in the plain area. The ground fissures identified in the area are mostly distributed along the main active faults and are consistent with the strikes of the faults [4,8]. The distribution of the land subsidence areas is obviously controlled by the faults, and the subsidence areas are mostly located where the active faults intersect. The development trend of the land subsidence is consistent with the strikes of the faults, and the uneven subsidence of the areas on both sides of the faults is very obvious [9,10]. In summary, active faults are the main potential disaster-causing geological features [10,11].

**Figure 2:**
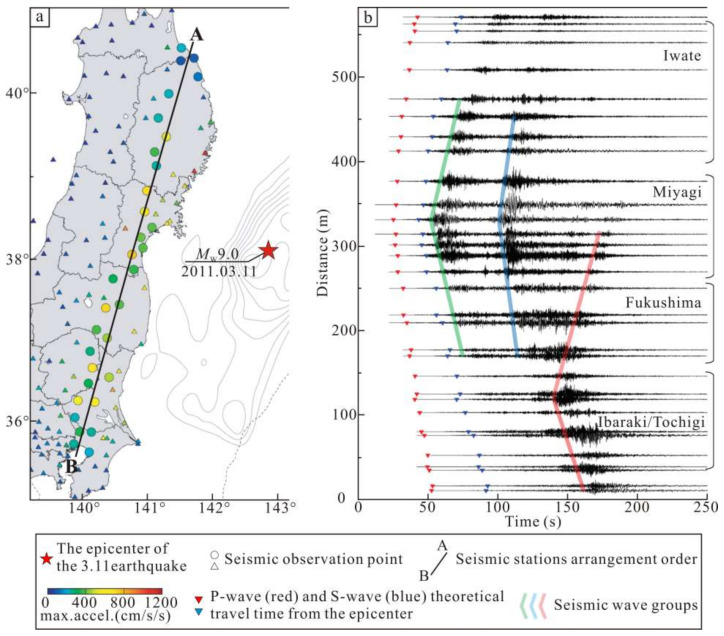
Acceleration seismograms of Tohoku-Oki 3.11 M9.0 earthquake. (**a**) Map showing the location of observed stations. (**b**) Acceleration seismograms at stations along a line from north to south [22].

Strong seismic activity can cause near-field and far-field stress changes, which has been confirmed by in situ stress measurements and real-time monitoring [12,13]. The Beijing Plain is located in the East China stress zone and North China stress zone [14]. Previous studies have shown that the subduction of the Pacific plate under the Eurasian plate is an important source of stress in North China [15,16]. The strong earthquake activity in this subduction zone has different degrees of influence on the in situ stress field and seismic activity in North China [17,18,19]. On 11 March 2011, the Tohoku-Oki M9.0 earthquake (hereinafter referred to as the 3.11 earthquake) occurred in the eastern sea of Japan (142.86° E, 38.10° N) (Figure 1). The acceleration seismograms of the 3.11 earthquake are shown in Figure 2. It caused significant near-field and far-field crustal deformation. Multi-beam bathymetric data from active source seismic exploration revealed that the earthquake caused the Japanese Trench area to move southeast by about 50 m [20,21]. The ground accelerations around the Miyagi area were over 500~1000 cm/s/s, and the largest PGA, 2933 cm/s/s, was recorded at the K-NET Tsukidate [22,23]. In the range of 0~1000 km from the epicenter, the range of measured PGDs was from 1 cm to almost 6 m for the Tohoku-Oki, Japan [24]. Coseismic displacement measurements based on global positioning system (GPS) monitoring revealed that the 3.11 earthquake produced a nearly EW-trending tensile effect in eastern China, with a maximum coseismic horizontal displacement of 35 mm [25,26,27]. Sun, et al. (2015) [28] analyzed the groundwater level monitoring data in Changping, Beijing, and found that the water level changes near the monitoring wells exhibited a coseismic response to the 3.11 earthquake. In addition, due to the earthquake, the permeability of the aquifers near the monitoring wells increased significantly. Wang, et al. (2013) [29] extrapolated that the 3.11 earthquake may have stretched the Tancheng-Yingkou segment of the Tan-Lu fault zone and the Changbai Mountain volcano for decades based on a post-earthquake creep model and GPS observation data for one year after the earthquake. Hao and Zhu (2020) [30] analyzed GPS observation data from 1999 to 2017 and concluded that the post-earthquake relaxation and deformation in northeastern China and the Shandong Peninsula would be sustained for 1 to 11 years after the 3.11 earthquake. In summary, the 3.11 earthquake had a huge impact on the tectonic environment in eastern China, and this impact may still persist.

The change in the regional in situ stress field is the most direct cause of the fault activity in this region [31,32]. Therefore, the stability of the active faults in the region should be studied in combination with the characteristics of and changes in the in situ stress field. The predecessors have carried out much work in this area [31,32,33,34,35]. Townend and Zoback (2004) [36] calculated the friction coefficient of the San Andreas fault based on the in situ stress measurement data, and the results showed that the friction coefficient was about 0.6 in the shallow part of the borehole and about 0.2 in the deep part. Tan, et al. (2014) [37] found that the present tectonic activity is abnormal along the zone from Tangshan to Luanxian to Changli through the comparative analysis of the in situ stress measurement and real-time monitoring data of different key tectonic locations around Beijing and the comprehensive study of the seismic background of Tangshan and its surrounding areas. Feng, et al. (2015) [38] used the in situ measurement and monitoring data to calculate the stress state in different periods at the Shisanling borehole drilled at Changping, Beijing, and discussed the activity of the Nankou Piedmont fault based on the Mohr–Coulomb failure criterion and Byerlee’s law. Chen, et al. (2019) [39] analyzed the faults activity in the Xuefeng Mountain area based on the measured in situ stress data and the Mohr–Coulomb failure criterion and considered that the faults in this area are in a stable state. Through literature research, it can be seen that the current fracture stability analysis using in situ stress data is mainly based on the Mohr–Coulomb failure criterion and Byerlee’s law to calculate the friction coefficient of the fault plane and judge the fault stress accumulation level. The existing studies on the impact of the 3.11 Earthquake on the tectonic environment and faults activity in the Beijing Plain are mainly based on GPS observation data, which can only judge the adjustment process of the in situ stress field and fault stability from the trend. No systematic study of the dynamic adjustment of the in situ stress field of the Beijing Plain after the 3.11 earthquake and the current characteristics of the in situ stress field has been conducted, and thus, the impact of the dynamic adjustment of the in situ stress field on the stability of the main active faults in the plain area is not clearly understood.

In order to improve the geological safety level in the capital region, Tan, et al. (2020) [40,41] carried out the deep borehole in situ stress measurements and constructed a real-time in situ stress monitoring station in Changli, Hebei, in 2009. The Changli piezomagnetic stress monitoring station began to continuously record monitoring data on 15 December 2011. It completely recorded the dynamic adjustment process of the in situ stress field in the capital region after the 3.11 earthquake, providing an in situ stress monitoring basis for seismic geology and other related research.

In order to reveal the current state of the in situ stress field of the Beijing Plain and to evaluate the slip potential of the main active faults. For the first time, we used the in situ stress monitoring data to study the adjustment process of the in situ stress field and the faults stability in the Beijing Plain after the 3.11 Earthquake. We first determined the initial in situ stress field before the 3.11 earthquake based on existing in situ stress measurement data collected before the earthquake. Second, we analyzed the dynamic adjustment of the in situ stress field using real-time monitoring data from the Changli monitoring station from November 2011 to September 2019. Third, we quantitatively analyzed the stability of the main active faults in the plain area in each adjustment stage of the in situ stress field according to the Mohr–Coulomb failure criteria. Finally, we used the FSP v.1.0 software package to assess the slip probability of the main active faults under the influence of the uncertainty (or error) of the fault attribute parameters and the in situ stress field. The results of this study provide scientific support for ensuring the urban geological security of the capital region and provide a reference for exploring the application of real-time in situ stress monitoring in tectonic stability evaluation.

The FSP v.1.0 software package, which was developed by the Stanford Center for Induced and Triggered Seismicity of Stanford University, California, America and is a freely available tool that allows users to estimate the changes in the fault slip potential in a given area [33].

## 2. Geologic Setting

**Figure 3:**
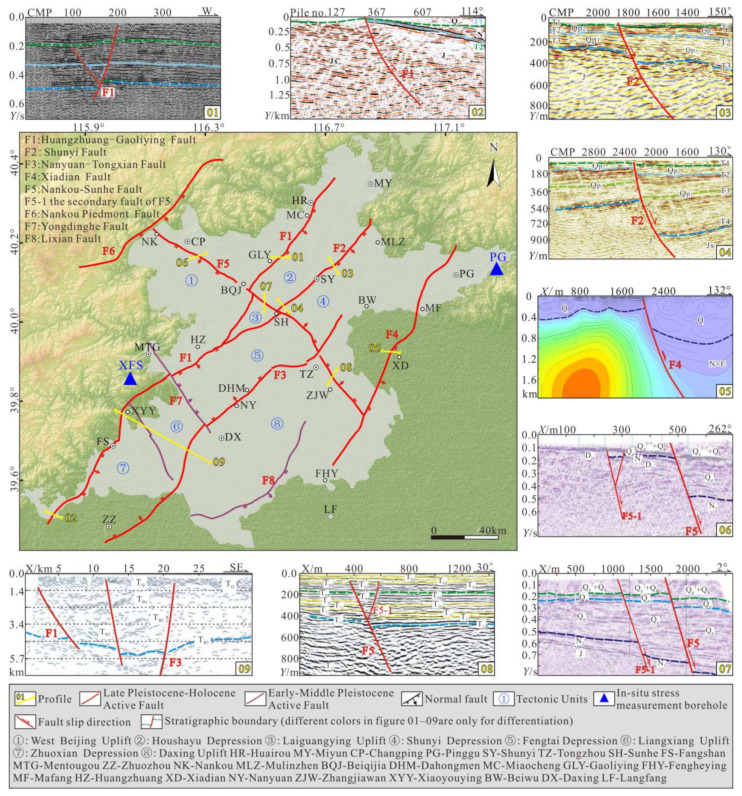
Spatial distribution of main active faults in the Beijing Plain. (01–02) The sections of seismic reflections across the Huangzhuang-Gaoliying Fault. (03–04) The sections of seismic reflections across the Shunyi Fault. (05) The section of resistivity inversion across the Xiadian Fault. (06–08) The sections of seismic reflections across the Nankou-Sunhe Fault. (09) The sections of seismic reflection across the southern Beijing Plain.

The Late Pleistocene–Holocene active faults in the Beijing Plain mainly include the Huangzhuang-Gaoliying fault, the Shunyi fault, the Nanyuan-Tongxian fault, the Xiadian fault, and the Nankou-Sunhe fault. Many scholars have carried out extensive research on the main active faults using geophysical, trenching, and drilling methods, and they have basically identified the geometric and kinematic characteristics of each active fault (Figure 3). A brief introduction to each active fault is provided below.

(1) The Huangzhuang-Gaoliying fault (F1) is a large-scale concealed normal fault that crosses the urban area of Beijing. The fault starts in Laishui (LS) County in the south and extends through Xiaoyouying (XYY), Huangzhuang (HZ), Gaoliying (GLY), and Miaocheng (MC) to the north of Huairou (HR), with a total length of about 132 km. The strike of this fault is NNE-NE, the dip is SE, and the dip angle is 63–67° [41,42]. The fault can be divided into three sections from north to south: the Miaocheng-Gaoliying section (the northern section), the Huangzhuang section (the middle section), and the Laishui section (the southern section), the northern and middle sections are bounded by the Nankou-Sunhe fault, and the middle section and southern section are roughly bounded by Xiaoyouying. The northern section is 40 km long and controls the Huairou and Shunyi depressions. This section was most recently active in the Holocene. The middle section is 52 km long and was active in the Middle and Late Pleistocene. The southern section is about 40 km long and has not been active in the Quaternary [43].

(2) The Shunyi fault (F2) is a concealed normal fault, which starts in the vicinity of Sunhe (SH) in the south and crosses through Shunyi (SY) City to Mulin Town (MLZ), i.e., trending NE, with a total length of about 35.6 km. It can be divided into two sections, northern and southern sections, with the Nankou-Sunhe fault as the boundary between the two sections. The strike of this fault is NNE-NE, the dip is SE, and the dip angle is 60–70° [7]. The shallow seismic detection results show that the upper breakpoint of the Shunyi fault is near the surface, indicating that the Shunyi fault is a Holocene active fault. In addition, the seismic activity on the Shunyi fault has increased in recent years, resulting in cracks in building walls and ground dislocation along the fault zone [44,45]. It indicates that this fault is still active and has creep characteristics.

(3) The Nanyuan-Tongxian fault (F3) is a concealed normal fault, which starts in Zhuozhou (ZZ) in the south and crosses through Nanyuan (NY) and Dahongmen (DHM) to Beiwu (BW), with a total length of about 78.6 km. The strike of this fault is 35–50°, the dip is NW, and the dip angle is about 80°. Drilling research has revealed that the Nanyuan-Tongxian fault is a Late Pleistocene active fault [4,46].

(4) The Xiadian fault (F4) is a concealed normal fault, which starts in Mafang (MF) in the north and passes through Xiadian (XD) to Fengheying (FHY) with a total length of about 85.1 km. The strike of this fault is 40–60°, the dip is SE, and the dip angle is about 65°. The Xiadian fault triggered the 1679 Sanhe-Pinggu *M*8.0 earthquake, and the steep ridge formed by the earthquake can still be seen today. This indicates that the Xiadian fault is a Holocene active fault [47,48].

(5) The Nankou-Sunhe fault (F5) is a concealed counterclockwise twisting normal fault, which starts at the Nankou piedmont fault in the northwest, crosses through Nankou (NK), Beiqijia (BQJ), Sunhe (SH), and Zhangjiawan (ZJW), and ends at the Xiadian fault, with a total length of about 80 km. The strike of this fault is 310–330°, and the dip angle is 65–74°. According to the fault occurrence, the Nankou-Sunhe fault can be divided into three sections, which are bounded by Beiqijia and Sunhe. The northwestern section of the fault dips to the southwest, while the middle section dips to the northeast, and there is little difference in the strikes and the dip angles of these two sections. Compared with the northwestern and middle sections, the southeastern section of the fault has a slightly different strike, and it is inclined to the northeast. The latest activity time in the northwestern and middle sections occurred in the Holocene–Late Pleistocene, and the latest activity in the southeastern section occurred slightly earlier (Late Middle Pleistocene) than that in the previous two sections [4].

## 3. Materials and Methods

### 3.1. Principle of Piezomagnetic Stress Monitoring

The essential part of a piezomagnetic stress monitoring system is the piezomangnetic stress gauge, which was designed based on the principle of magnetostriction. That is, the lengths and volumes of permalloy ferromagnetic materials, such as ferro-nickel iron, change when magnetized. These phenomena are referred to as linear magnetostriction and volume magnetostriction. In addition, mechanical deformation also changes the permeability of ferromagnetic materials, which is called the piezomagnetic effect [49]. The relative permeability change and stress have the following relationship:

(1) $$\frac{\Delta \mu }{\mu }=\frac{2\lambda }{\beta^{2}}\mu \sigma$$

where *μ* is the permeability, ∆*μ* is the change in the permeability, *β* is the saturated intensity of the magnetization, *λ* is the saturated magnetostriction coefficient, and *σ* is the stress that acts on the material. Usually, when the permalloy ferromagnetic materials are in the magnetized state, we choose the straight part of the magnetization curve, and therefore, this formula can be used to describe the relationship between the magnetostriction and stress acting on the ferromagnetic materials.

**Figure 4:**
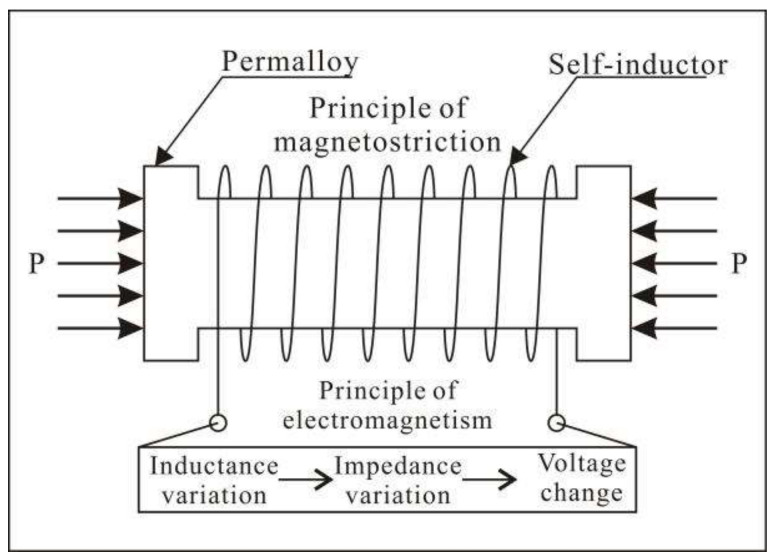
Principles of piezomagnetic stress monitoring.

The piezomangnetic stress gauge is mainly composed of a mandrel made of a special permalloy ferromagnetic material (iron–nickel alloy) and a self-inductor wound around it (Figure 4) [40]. When an external force acts on the ferromagnetic material, the permeability will change, which will cause the inductance of the coil to change, and thus, the impedance value of the coil will change, and finally the voltage value of the coil will change. By keeping the current constant and monitoring the change in the coil voltage in real time, and according to the calibration curve of the indoor surrounding pressure and voltage, the stress change acting on the measuring element in the monitoring probe can be obtained.

### 3.2. Fault Sliding Instability Criterion

The evaluations of fault stability are mainly based on the Mohr–Coulomb failure criterion and Byerlee’s law. Fault slip is a process in which the shear stress on the fault layer gradually accumulates and exceeds the anti-sliding resistance under the action of the in situ stress field, resulting in sliding instability [50,51]. Numerous studies have shown that this mechanical process follows the Mohr–Coulomb failure criterion [52,53,54]:

(2) $$\tau \geq \mu \left(\sigma_{n}-P_{0}\right),\;$$

where *τ* is the shear stress of the fault plane, *μ* is the friction coefficient of the fault plane, *σ_n_* is the normal stress of the fault plane, and *P*_0_ is the pore water pressure. Studies have shown that in low-permeability rocks on the shallow surface of the crust, the pore pressure is roughly equal to the hydrostatic pressure, so in this paper, the pore pressure was assumed to be approximately equal to the hydrostatic pressure [55].

Byerlee (1978) [56] summed up the results of numerous rock mechanics experiments and considered that *μ* of crustal rocks is 0.6–1.0, except for a few rocks. In situ stress measurements conducted by the researchers proved that *μ* measured in the laboratory is suitable for the crust [34,57,58,59].

Combining the Mohr–Coulomb fracture criterion and Byerlee’s law, we know that the level of stress accumulation on the fault plane can be determined by comparing *τ*/(*σ_n_* − *P*_0_) and *μ*; therefore, an appropriate *μ* value is the key to evaluating fault stability. The friction coefficient is influenced by many factors, including stress, temperature, and fault material [60]. In this study, only the static friction coefficient was considered.

### 3.3. Least-Squares Solution Algorithm for Principal Stress Magnitude and Orientation Based on Monitoring Data

**Figure 5:**
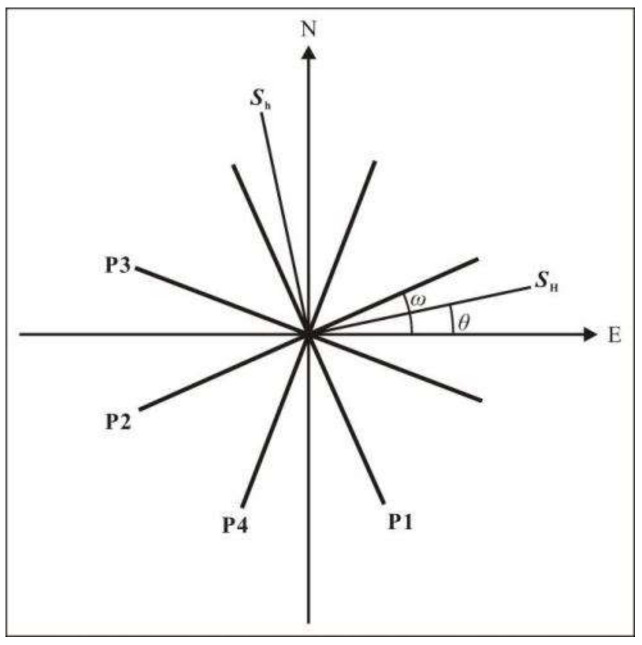
Diagram showing the spatial relationships between the monitoring azimuth and principal stress direction.

A Cartesian coordinate system (XOY) was established. In this coordinate system, the positive direction on the *x*-axis is due east, and the positive direction on the *y*-axis is due north. The angles between the component probes (P1–P4) are 45° to each other. The angle between P2 and the *x*-axis is *ω*, and the angle between the *S_H_* orientation and the *x*-axis is *θ* (Figure 5).

The normal stress changes in the four monitoring directions (P1–P4) in a certain stage are recorded as ∆*σ*_1_, ∆*σ*_2_, ∆*σ*_3_, and ∆*σ*_4_, and the resulting changes in the plane stress components in the XOY coordinate system are recorded as ∆*σ_xx_*, ∆*σ_yy_*, and ∆*τ_xy_*. Thus, according to the two-dimensional plane stress tensor transformation formula [31], the following four equations can be obtained:

(3) $$\left\{\begin{array}{l} \Delta \sigma_{1}=\frac{1}{2}\left(\Delta \sigma_{xx}+\Delta \sigma_{yy}\right)+\frac{1}{2}\left(\Delta \sigma_{xx}-\Delta \sigma_{yy}\right)\cos2\omega +\Delta \tau_{xy}\sin2\omega \\ \Delta \sigma_{2}=\frac{1}{2}\left(\Delta \sigma_{xx}+\Delta \sigma_{yy}\right)-\frac{1}{2}\left(\Delta \sigma_{xx}-\Delta \sigma_{yy}\right)\sin2\omega +\Delta \tau_{xy}\cos2\omega \\ \Delta \sigma_{3}=\frac{1}{2}\left(\Delta \sigma_{xx}+\Delta \sigma_{yy}\right)-\frac{1}{2}\left(\Delta \sigma_{xx}-\Delta \sigma_{yy}\right)\cos2\omega -\Delta \tau_{xy}\sin2\omega \\ \Delta \sigma_{4}=\frac{1}{2}\left(\Delta \sigma_{xx}+\Delta \sigma_{yy}\right)+\frac{1}{2}\left(\Delta \sigma_{xx}-\Delta \sigma_{yy}\right)\sin2\omega -\Delta \tau_{xy}\cos2\omega \end{array}\right.$$

In the above equations, ∆*σ*_1_, ∆*σ*_2_, ∆*σ*_3_, ∆*σ*_4_, and *ω* are all known quantities, from which the least-squares solutions of ∆*σ_xx_*, ∆*σ_yy_*, and ∆*τ_xy_* can be obtained as follows:

(4) $$\left\{\begin{array}{l} \Delta \sigma_{xx}=\frac{\Delta \sigma_{1}+\Delta \sigma_{2}+\Delta \sigma_{3}+\Delta \sigma_{4}}{4}+\frac{\left(\Delta \sigma_{1}-\Delta \sigma_{3}\right)\cos2\omega -\left(\Delta \sigma_{2}-\Delta \sigma_{4}\right)\sin2\omega }{2}\\ \Delta \sigma_{yy}=\frac{\Delta \sigma_{1}+\Delta \sigma_{2}+\Delta \sigma_{3}+\Delta \sigma_{4}}{4}-\frac{\left(\Delta \sigma_{1}-\Delta \sigma_{3}\right)\cos2\omega -\left(\Delta \sigma_{2}-\Delta \sigma_{4}\right)\sin2\omega }{2}\\ \Delta \tau_{xy}=\frac{\left(\Delta \sigma_{1}-\Delta \sigma_{3}\right)\sin2\omega +\left(\Delta \sigma_{2}-\Delta \sigma_{4}\right)\cos2\omega }{2} \end{array}\right.$$

According to the two-dimensional plane stress tensor transformation formula [31], the plane stress components *σ^P^_xx_*, *σ^P^_yy_*, and *τ^P^_xy_* in the XOY rectangular coordinate system of the initial horizontal principal stress can be obtained. After superimposing with the XOY plane stress changes in this stage, the two-dimensional plane stress tensor conversion formula can be used to obtain the maximum and minimum horizontal principal stresses, and the direction of the maximum horizontal principal stress can be calculated as follows:

(5) $$\left\{\begin{array}{l} S_{H}=\frac{\left(\sigma_{xx}^{P}+\Delta \sigma_{xx})+(\sigma_{yy}^{P}+\Delta \sigma_{yy}\right)}{2}+\frac{\sqrt{(\sigma_{xx}^{P}+\Delta \sigma_{xx})-(\sigma_{yy}^{P}+\Delta \sigma_{yy})+4{\left(\tau_{xy}^{P}+\Delta \tau_{xy}\right)}^{2}}}{2}\\ S_{h}=\frac{\left(\sigma_{xx}^{P}+\Delta \sigma_{xx})+(\sigma_{yy}^{P}+\Delta \sigma_{yy}\right)}{2}-\frac{\sqrt{(\sigma_{xx}^{P}+\Delta \sigma_{xx})-(\sigma_{yy}^{P}+\Delta \sigma_{yy})+4{\left(\tau_{xy}^{P}+\Delta \tau_{xy}\right)}^{2}}}{2}\\ \tan2\theta =\frac{2(\tau_{xy}^{P}+\Delta \tau_{xy})}{\left(\sigma_{xx}^{P}+\Delta \sigma_{xx})-(\sigma_{yy}^{P}+\Delta \sigma_{yy}\right)} \end{array}\right.$$

## 4. Results

### 4.1. In Situ Stress Field in the Shallow Crust of the Beijing Plain before the 3.11 Earthquake

Before the 3.11 earthquake, the research on the measured stress field of the Beijing area was insufficient. The main research was carried out after the 1976 Tangshan *M*7.8 earthquake, and the in situ stress measurement depth was generally shallow (≤300 m). Tan, et al. (2020) [40,61] carried out in situ stress measurements in two deep holes (600–800 m) in Pinggu (PG) and Xifengsi (XFS) in 2008 and 2010, respectively. The Pinggu borehole (Figure 3) was located in the north section of the Xiadian fault, with a depth of 600.47 m, and the lithology was predominantly Yanshanian granite (0–413.13 m) and Mesoproterozoic limestone (413.13–600.47 m). The Xifengsi borehole (Figure 3) was located in the middle section of the Huangzhuang-Gaoliying fault, with a depth of 800.32 m, and the lithology was predominantly Triassic and Jurassic sandstones. The in situ stress measurements were collected using the hydraulic fracturing method, which is one of the two major in situ stress estimation methods suggested by the International Society for Rock Mechanics (ISRM) [62]. This method has the advantages of simple operation, large measurement depth, reliable data, and repeatable tests [35,63,64,65]. 

**Figure 6:**
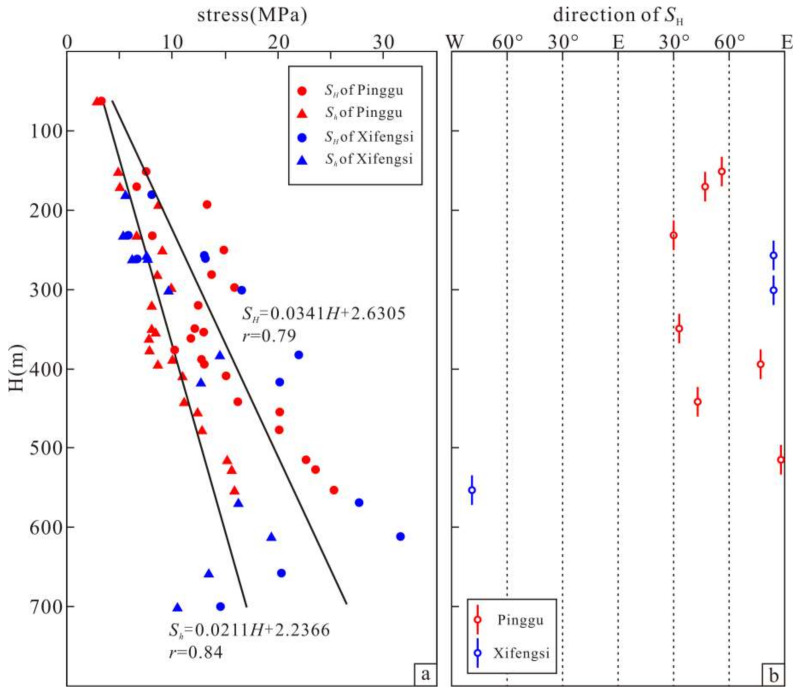
Variations in the measured horizontal stress and its orientation with depth in the Beijing Plain before the Tohoku-Oki 3.11 M9.0 earthquake. (**a**) The magnitudes of two principal stresses with the depth. (**b**) The orientations of the maximum horizontal principal stress.

The hydraulic fracturing measurement results of the two boreholes are presented in Figure 6. The in situ stress measurement results in the measurement depth range of the Pinggu borehole show that the maximum horizontal principal stress (*S_H_*) was 3.31–25.31 MPa, and the minimum horizontal principal stress (*S_h_*) was 2.88–15.89 MPa. The in situ stress measurement results in the measurement depth range of the Xifengsi borehole show that the *S_H_* was 5.84–31.60 MPa and the *S_h_* was 5.36–19.36 MPa. Both borehole measurements show that the surface layer of the crust in the Beijing Plain was dominated by horizontal tectonic stress. Based on the in situ stress measurement data for the two boreholes, the relationship between *S_H_* and *S_h_* in the Beijing Plain area with depth was calculated via linear regression, and the results are shown in Equation (6):

(6) $$\left\{\begin{array}{l} S_{H}=0.0341H+2.6305\begin{array}{cc} & \left(r=0.79,n=35\right) \end{array}\\ S_{h}=0.0211H+2.2366\begin{array}{cc} & \left(r=0.84,n=35\right) \end{array} \end{array}\right.,$$

where *H* is the measurement depth, *r* is the correlation coefficient, and *n* is the number of fitted samples.

Equation (6) and Figure 6 reveal that the in situ stress of the shallow surface crust in Beijing Plain increases with increasing depth, and the maximum and minimum horizontal principal stress gradients are 0.0341 MPa/m and 0.0211 MPa/m, which are slightly greater than the average level in North China [66].

The measurement results of the maximum horizontal principal stress orientation (*S_H_* orientation) in the Pinggu borehole and Xifengsi borehole show that the orientation of *S_H_* in the shallow part of the borehole (≤250 m) is mainly NE (N 30–56° E), while the orientation of *S_H_* in the deep part of the borehole (≥250 m) is NEE (N 77–88° E). The variations in the measurement results with depth are mainly affected by the topography [67]. The results of the source mechanism inversion [68], numerical simulation of the tectonic stress field [69], and analysis of the fault sliding data [70] have all revealed that the orientation of *S_H_* in the Beijing Plain was NEE–EW before the 3.11 earthquake. In summary, the dominant orientation of the *S_H_* in the shallow crust of the Beijing Plain should be in the NEE direction (N 77–88° E).

### 4.2. In Situ Stress Monitoring Data

The Changli piezomagnetic stress monitoring station (119.15° E, 39.74° N) is located in the large Yanshanian granite body on the north side of the intersection of the Tan-Lu fault zone and the Zhangjiakou-Bohai fault zone. It is one of the most ideally located in situ stress monitoring stations in North China [40,61] (Figure 1). The monitoring drilling depth is 500.46 m. The lithology of the stratum within the drilling depth range is mainly medium and coarse-grained granite. The station uses a new type of four-component piezomagnetic stress monitoring system. The monitoring instrument is placed at a depth of 100.0 m. The monitoring directions of the four probes are N 336° W (P1), N 66° E (P2), N 291° W (P3), and N 21° E (P4). The station began to record data on 7 December 2011, and it completely recorded the adjustment and changes of the in situ stress field in North China after the 3.11 earthquake.

**Figure 7:**
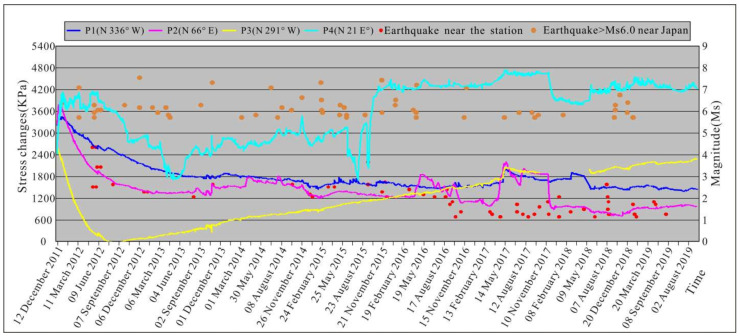
Relative changes in the in situ stress at the Changli piezomagnetic stress monitoring station from December 2011 to September 2019.

The in situ stress data recorded by the Changli piezomagnetic stress monitoring station from 15 December 2011 to 8 September 2019 are shown in Figure 7. The stress curve of each probe represents the relative change in the normal stress in the corresponding monitoring direction, and the fluctuations of the curve reflect the dynamic adjustment of the regional in situ stress field. According to the changes in the in situ stress monitoring curve, the dynamic adjustment of the in situ stress field after the 3.11 earthquake can be divided into five stages: stage 1 (November 2011 to June 2012), stage 2 (June 2012 to May 2013), stage 3 (May 2013 to June 2015), stage 4 (June 2015 to December 2015), and stage 5 (December 2015 to September 2019).

### 4.3. Analysis of the Dynamic Change in the In Situ Stress Field after the 3.11 Earthquake

According to Equation (6), the initial value of *S_H_* at a depth of 100.0 m (descending depth of the monitoring instrument) was 6.04 MPa, and the initial value of *S_h_* was 4.35 MPa. The relative changes in the normal stress at the end of the five stages were extracted, and the *S_H_* and *S_h_* at each time node were calculated using Equations (4) and (5), which were then used to analyze the dynamics of the in situ stress field of the Beijing Plain after the 3.11 earthquake. The calculation results are presented in Table 1.

In stage 1 (11 March 2011–6 June 2012), the variation characteristics of the stress curves in each monitoring direction were as follows. The stress value in the P4 (N 21° E) direction increased rapidly and was in a high-value state, while the stress value in the P3 (N 291° W) direction exhibited a rapid decrease. The stress in the P1 (NW 336°) direction and P2 (N 66° E) direction increased rapidly for a short period of time and then decreased slowly. The calculated magnitude and direction of the principal stress at the end of stage 1 revealed that the maximum horizontal principal stress in the Beijing Plain was 6.04–6.35 MPa, which was slightly higher than that before the 3.11 earthquake, while the minimum horizontal principal stress value was 2.71–3.02 MPa, which was significantly lower than that before the 3.11 earthquake. The direction of the maximum horizontal principal stress was deflected from the NEE direction to the NNE direction. Stage 1 was the early post-earthquake period. The strong subduction of the Pacific plate has produced a nearly EW-trending extensional effect in North China and Northeast China. The regional tectonic environment changed from nearly EW compression to nearly SN compression within a short period of time after the 3.11 earthquake. This change inevitably led to deflection of the orientation of the maximum horizontal principal stress in the Beijing Plain. In addition, the coseismic displacement field of the 3.11 earthquake showed that the east-west displacement in North China was significant in the early post-earthquake period, while the north-south displacement was not obvious [25,26]. This situation caused the minimum horizontal principal stress (SEE) to decrease significantly and the maximum horizontal principal stress (NNE) to change little in the plain area.

In stage 2 (6 June 2012–4 May 2013), the variation characteristics of the stress curve in each monitoring direction were as follows. After 6 June 2012, the stress value in the P4 (N 21° E) direction began to gradually decrease, while that in the P3 (N 291° W) direction changed from a continuous decrease to a slow increase. The calculated magnitude and direction of the principal stress at the end of stage 2 revealed that the maximum horizontal principal stress in the Beijing Plain area was 4.17–4.49 MPa, which was significantly lower than that during stage 1, the minimum horizontal principal stress was 2.86–3.17 MPa, and the orientation of the maximum horizontal principal stress changed from NNE to N 42.9°–46.4° E. In stage 2, the nearly EW tensile effect in the Beijing Plain area changed from strong to weak, and the regional tectonic environment began to gradually adjust back to the pre-earthquake state. The GPS monitoring data revealed that the Nankou-Sunhe fault underwent dextral compressional movement for about a year after the earthquake, and then it quickly returned to its previous left-lateral extensional movement [70]. This also indicates that the nearly EW-trending tensile stress environment in the Beijing Plain area lasted for about one year, and then it began to adjust to the nearly EW-trending compressive stress environment. The larger decrease in the maximum horizontal principal stress value was mainly due to the stress release caused by the coseismic displacement in the nearly EW direction.

In stage 3 (4 May 2013–16 June 2015), the variation characteristics of the stress curve in each monitoring direction were as follows. The monitoring values in the P2 (N 66° E) direction and P3 (N 291° W) direction gradually increased, and that in the P4 (NE 21°) direction fluctuated within a certain range after a short period of sharp increase. The value in the P1 (N 336° W) direction basically remained stable. The calculated magnitude and direction of the principal stress at the end of stage 3 revealed that the maximum horizontal principal stress in the Beijing Plain was 5.00–5.32 MPa, the minimum horizontal principal stress was 3.04–3.36 MPa, and the orientation of the maximum horizontal principal stress was N 40.8°–42.1° E. In stage 3, under the continuous action of the nearly east–west extrusion, the stress began to accumulate gradually in the Beijing Plain area. The principal stress increased, but the direction of the maximum horizontal principal stress did not change much. This indicates that the in situ stress in the Beijing Plain area had not yet adjusted back to the pre-earthquake state.

In stage 4 (16 June 2015–15 December 2015), the variation characteristics of the stress curve in each monitoring direction were as follows. The monitoring values in the P1 (N 336° W) direction and P4 (N 21° E) direction decreased sharply and then gradually increased. That in the P4 (N 21° E) direction fluctuated more widely, and the recovery rate of the monitoring value in the P2 (N 66° E) direction remained basically unchanged. The calculated magnitude and direction of the principal stress at the end of stage 4 revealed that the maximum horizontal principal stress in the Beijing Plain was 5.70–6.01 MPa, the minimum horizontal principal stress was 2.96–3.28 MPa, and the orientation of the maximum horizontal principal stress was N 32.4–33.5° E. It is inferred from the changes in the monitoring values that the subduction of the Pacific plate intensified during this period, which led to the strengthening of the nearly SN compression in North China. The violent fluctuation of the monitoring values in the P4 direction likely occurred in response to the Changli *M*_S_4.2 earthquake (118.81° E, 39.73° N) at 18:00 on 14 September 2015. In contrast to the end of stage 3, the principal stress value in the Beijing Plain area did not decrease at the end of stage 4, indicating that the stress accumulation of the in situ stress field in North China is still continuing.

In stage 5 (15 December 2015–8 September 2019), the variation characteristics of the stress curve in each monitoring direction were as follows. The stress value in the P4 (N 21° E) direction was relatively stable in the early stage, and then it slowly increased after October 2018. The value in the P3 (N 291° W) direction continued to increase slowly, and those in the P1 (N 336° W) and P2 (N 66° E) directions remained relatively stable. The calculated magnitude and direction of the principal stress at the end of stage 5 revealed that the maximum horizontal principal stress in the Beijing Plain was 5.30–5.62 MPa, the minimum horizontal principal stress was 3.61–3.93 MPa, and the orientation of the maximum horizontal principal stress was N 39.0–39.6° E. The analysis results show that the activity of the Pacific plate was relatively stable after December 2015, and the stress accumulation of the in situ stress field in the Beijing Plain area continued, but it had not yet recovered to the pre-earthquake level.

In summary, the 3.11 earthquake had a significant impact on the in situ stress field in the Beijing Plain. The regional tectonic environment experienced a dynamic change from nearly EW compression to nearly EW extension in a short period of time after the earthquake, and then it gradually returned to nearly EW compression. Currently, the in situ stress field is still adjusting back to the pre-earthquake state, but it had not yet reached the pre-earthquake level by the end of the study period.

### 4.4. Analysis of the Stability of the Main Active Faults in the Beijing Plain Area after the 3.11 Earthquake

#### 4.4.1. Simplified Model of the Main Active Faults

**Figure 8:**
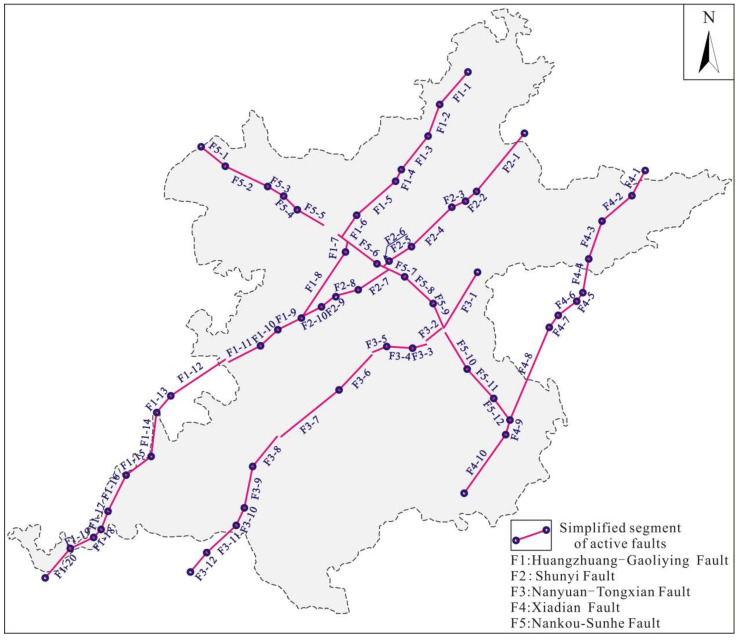
Simplified segmental features of the main buried faults in the Beijing Plain.

Due to the differences in the fault strike, the main active faults were simplified to facilitate analysis of the fault slip potential. The Huangzhuang-Gaoliying fault was divided into 20 subsections (F1-1–F1-20), the Shunyi fault was divided into 10 subsections (F2-1–F2-10), the Nanyuan-Tongxian fault was divided into 12 subsections (F3-1–F3-12), the Xiadian fault was divided into 10 subsections (F4-1–F4-10), and the Nankou-Sunhe fault was divided into 12 subsections (F5-1–F5-12) (Figure 8).

The simplification of faults causes an error in the strike, and existing studies have presented slightly different understandings of the strike and dip angles of the major faults. In order to fully consider the uncertainty of the fault strike and dip angle, the fault strike and dip angles of each subsection of the five faults were assigned uncertainties of ±5° and ±5°, respectively. The attribute parameters, including the strike, dip angle, and length, of each subsection are presented in Table 2.

#### 4.4.2. Selection of Critical Friction Coefficient

A large number of rock mechanics experiments have shown that the friction coefficient of brittle rock in friction equilibrium is between 0.6 and 1.0 [55,59]. When it comes to assessing the fault slip potential, an empirical friction coefficient of 0.6 used typically invoked as the critical value [33,71]. For example, Walsh and Zoback (2016) evaluated the injection-induced faulting instability and seismicity in northern and central Oklahoma using a friction coefficient of 0.6 [33]. Feng, et al. (2014) evaluated the stability of the faults in the northern part of Beijing using a friction coefficient of 0.6 [72]. As such, under the premise that it is difficult to directly determine the friction coefficient of the main hidden faults in the Beijing Plain, we temporarily selected 0.6 as the critical friction coefficient for evaluating the fault slip potential.

#### 4.4.3. Quantitative Evaluation of the Stability of the Main Active Faults after the 3.11 Earthquake

**Figure 9:**
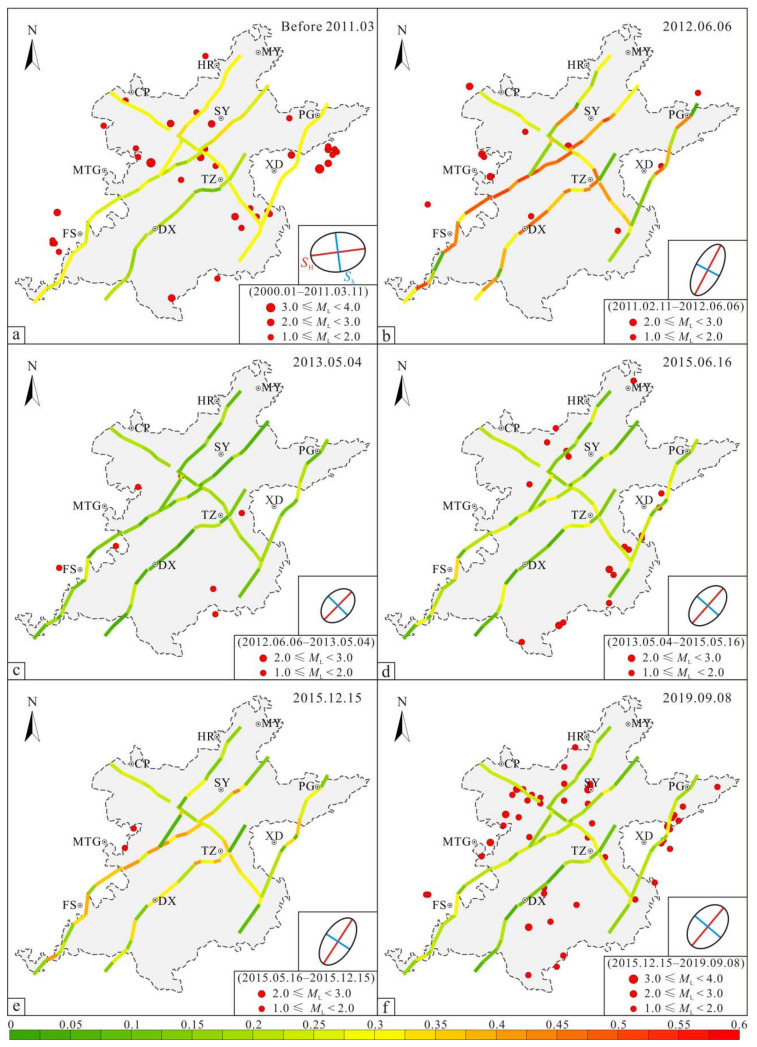
Results of the quantitative evaluation on the stability of the main active faults in the Beijing Plain. (**a**) Quantitative evaluation results of the fracture stability before the 3.11 earthquake. (**b**) Quantitative evaluation results of the fracture stability in stage 1. (**c**) Quantitative evaluation results of the fracture stability in stage 2. (**d**) Quantitative evaluation results of the fracture stability in stage 3. (**e**) Quantitative evaluation results of the fracture stability in stage 4. (**f**) Quantitative evaluation results of the fracture stability in stage 5.

After the 3.11 earthquake, the dynamic adjustment of the in situ stress field of the Beijing Plain caused changes in the stability of the faults. Based on the adjustment results of the post-earthquake in situ stress field in the Beijing Plain area (Table 1), we calculated the effective normal stress and shear stress along the main active faults [13], and we quantitatively assessed the stability of the main active faults based on the Mohr–Coulomb failure criteria (Equation (2)). Figure 9 presents the results. In this section, we focus on analyzing the tectonic stability of the main active faults in the Beijing Plain area before the 3.11 earthquake (Figure 9a) and in stage 5 (Figure 9f). In addition, we provide a brief overview of the dynamic adjustment of the tectonic stability of the faults from stage 1 to stage 4.

Before the 3.11 earthquake, the friction coefficient of each fault subsection under the action of the NEE-directed principal compressive stress field was calculated (Figure 9a). The *μ* values of the subsections of the Huangzhuang-Gaoliying fault (F1) were 0.24–0.33, with an average of 0.29. The northern section (F1-1–F1-6) had *μ* values of 0.31–0.33, with an average of 0.32; the middle section (F1-7–F1-14) had *μ* values of 0.24–0.30, with an average of 0.27; and the southern section (F1-15–F1-20) had *μ* values of 0.25–0.31, with an average of 0.29. This indicates that the activity on the northern section of the Huangzhuang-Gaoliying fault was greater than those on the middle and southern sections. The *μ* values of the subsections of the Shunyi fault (F2) were 0.17–0.34, with an average of 0.26. The northern section (F2-1–F2-6) had *μ* values of 0.24–0.34, with an average of 0.29, and the southern section (F2-7–F2-10) had *μ* values of 0.17–0.26, with an average of 0.22. This indicates that the activity on the northern section of the Shunyi fault was greater than that on the southern section. The *μ* values of the subsections of the Nanyuan-Tongxian fault (F3) were 0.10–0.22, with an average of 0.18. The *μ* values of the subsections of the Xiadian fault (F4) were 0.29–0.31, with an average of 0.30. The *μ* values of the subsections of the Nankou-Sunhe fault (F5) were 0.25–0.30, with an average value of 0.28. The *μ* values of the northwestern section (F5-1–F5-5) were 0.28–0.29; the *μ* values of the middle section (F5-6) were 0.25; and the *μ* values of the southeastern section (F5-7–F5-12) were 0.28–0.30, with an average of 0.29. This indicates that the southeastern and northwestern sections of the Nankou-Sunhe fault were more active than the middle section. In summary, before the 3.11 earthquake, the *μ* values of the subsections of the five main faults in the Beijing Plain did not exceed the critical friction coefficient of 0.6, and the stress accumulation was at a low to medium level. Among them, the Nankou-Sunhe fault, the Xiadian fault, and the Huangzhuang-Gaoliying fault were the most active, followed by the Shunyi fault, and the Nanyuan-Tongxian fault was the least active. The Beijing Plain area was divided using the Nankou-Sunhe fault as the boundary, and the activity of the faults in the northeastern region was greater than that of the faults in the southwestern region. The statistical results of the earthquakes in the Beijing Plain area from January 2000 to March 2011 correspond well with the results of the fault stability analysis. The earthquakes in the Beijing Plain area during this period were all microseisms, indicating that the faults in the plain area were active but weak in intensity. In addition, the earthquakes mainly concentrated in the northeastern part of the plain area and were mainly distributed along both sides of the Sunhe-Nankou fault.

After the 3.11 earthquake, due to the short-term adjustment of the principal stress direction from NEE to NNE in stage 1 (Figure 9b), the activity in the central and southern sections of the Huangzhuang-Gaoliying fault, the Shunyi fault, and the Nanyuan-Tongxian fault significantly increased. The calculated *μ* values of the subsections of the five main faults were concentrated within 0.28–0.51. The northern section of the Huangzhuang-Gaoliying fault and the Xiadian fault exhibited increased activity in individual subsections, but their overall activity decreased. The activity of the northwestern and middle sections of the Nankou-Sunhe fault weakened, while the activity of the southeastern section increased. In stage 2 (Figure 9c), under the stress release caused by the post-earthquake coseismic displacement and the gradual return of the regional tectonic environment to the pre-earthquake state, the *μ* values of the five main faults decreased significantly, and the *μ* values of the subsections were 0.03–0.25. This indicates that the activity on the major faults weakened in stage 2, and the tectonic environment in the plain area tended to be stable during this short period of time. From stage 3 to stage 4 (Figure 9d,e), the tectonic stress field of the Beijing Plain began to accumulate gradually, and the principal stress value gradually increased. In addition, due to the subduction strength of the Pacific plate, the principal stress direction shifted slightly toward the NE, and the calculated cross-sectional *μ* values of the Huangzhuang-Gaoliying fault, the Shunyi fault, the Nanyuan-Tongxian fault, and the Xiadian fault fluctuated greatly. Moreover, the earthquake statistics from May 2013 to December 2015 revealed that the microseisms were mainly distributed along the NE-trending faults, indicating that under the action of the NE-trending tectonic stress field, the activity on the NE-trending faults in the Beijing Plain was relatively strong.

The quantitative evaluation of the present-day fracture stability in the Beijing Plain area is shown in Figure 9f (stage 5). Under the action of the NE-trending principal compressive stress field, the *μ* values of the subsections of the Huangzhuang-Gaoliying fault (F1) were 0.14–0.28, with an average of 0.21. The *μ* values of the northern section (F1-1–F1-6) were 0.16–0.24, with an average value of 0.19, and the *μ* values of the middle section (F1-7–F1-14) were 0.14–0.28, with an average value of 0.21; this indicates that the activity in the middle and southern sections of the Huangzhuang-Gaoliying fault was greater than that in the northern section. The *μ* values of the subsections of the Shunyi fault (F2) were 0.12–0.29, with an average of 0.21. The northern section (F2-1–F2-6) had *μ* values of 0.12–0.29, with an average of 0.19, and the southern section (F2-7–F2-10) had *μ* values of 0.17–0.27, with an average of 0.22. This indicates that the activity in the southern section of the Shunyi fault was greater than that in the northern section. The *μ* values of the subsections of the Nanyuan-Tongxian fault (F3) were 0.06–0.24, with an average of 0.15. The *μ* values of the subsections of the Xiadian fault (F4) were 0.15–0.28, with an average of 0.22. The *μ* values of the subsections of the Nankou-Sunhe fault (F5) were 0.17–0.28, with an average of 0.22. The *μ* values of the northwestern section (F5-1–F5-5) were 0.23–0.24; the *μ* values of the middle section (F5-6) were 0.17; and the *μ* values of the southeastern section (F5-7–F5-12) were 0.25–0.28, with an average of 0.26. This indicates that the southeastern and northwestern sections of the Nankou-Sunhe fault were more active than the middle section. In summary, under the action of the present in situ stress field, the *μ* values of the subsections of the five main faults in the Beijing Plain area did not exceed the critical friction coefficient of 0.6, and the stress accumulation level was lower than before the 3.11 earthquake. Based on the analysis of the spatial distribution characteristics of the earthquakes in this region, the activity on the central and southern sections of the Huangzhuang-Gaoliying fault, the Xiadian fault, and the northwestern and southeastern sections of the Nankou-Sunhe fault was relatively strong.

The results of the quantitative analysis of the stability of the main faults in the Beijing Plain also revealed that the *μ* values of the main active fault sections in each stage did not exceed 0.6, but the micro-earthquakes continued. At the end of stage 1, the *μ* value of the F1-19 subsection of the Huangzhuang-Gaoliying fault and that of the F2-3 subsection of the Shunyi fault were the largest (0.52). This indicates that the boundary friction coefficients of the fault sections were less than 0.6. In each adjustment stage, the Nanyuan-Tongxian fault section had the smallest *μ* value, among which the *μ* values of the subsections before the earthquake (Figure 9a) were 0.10–0.22, and there were no earthquakes near the fault. The *μ* values of the subsections at the end of the study period (Figure 9f) were 0.06–0.24, and there were occasional earthquakes near the fault, so it can also be inferred that the critical friction coefficient of the main hidden faults in the Beijing Plain is 0.22 ≤ *μ* ≤ 0.52.

#### 4.4.4. Probabilistic Analysis of Slip on the Main Active Faults

The quantitative evaluation of the fault stability does not consider the parameter error value; however, each parameter is not a fixed value, and there is a certain level of uncertainty (or error). As shown in Table 1, the *S_H_*, *S_h_*, and *S_H_* orientations are uncertain. Because the gravitational loading *S*_V_ was estimated using the unit weight, we did not consider its uncertainty. As shown in Table 2, the strikes and dip angles of the fault subsections have an error range of 5°. The groundwater level in the Beijing Plain is 25 ± 5 m, so the pore water pressure is also uncertain. The fracture stability shows that the critical fault coefficient is between 0.22 and 0.52, with an average value of 0.37 ± 0.15. The above details are shown in Table 3.

**Figure 10:**
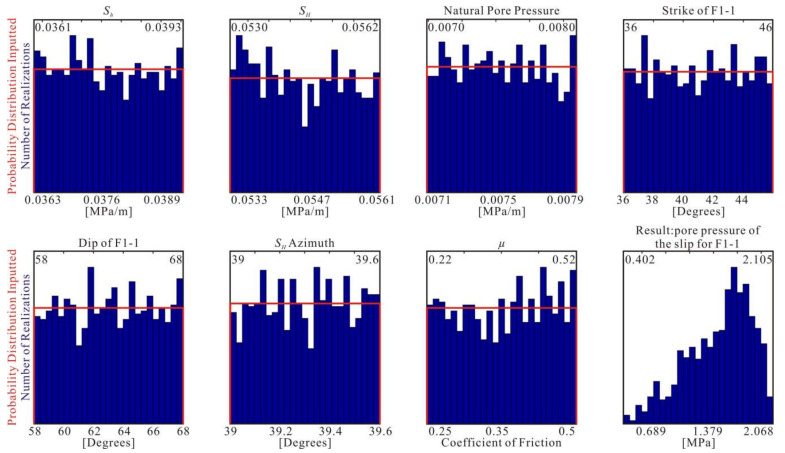
Probability distributions of the in situ stress field and the characteristic parameters of mapped fault subsection F1-1. The uniform distribution of each parameter’s uncertainty is plotted in red, and the bounds are labeled in the upper corners of each plot. Each blue histogram shows the actual distribution of each random input after many Monte Carlo iterations.

We used the FSP v.1.0 software package to evaluate the possibility of sliding instability occurring on the major active faults in the Beijing Plain area [33,71,72]. To minimize the uncertainties, we evaluated the possibility of slipping along these faults in response to an increase in the pore pressure using the Monte Carlo simulation method to randomly sample the specified uniform uncertainty distributions of the input parameters. Taking subsection F1-1 of the Huangzhuang-Gaoliying fault as an example, the resampling of the probabilistic distribution of the model parameters for subsection F1-1 is shown in Figure 10.

**Figure 11:**
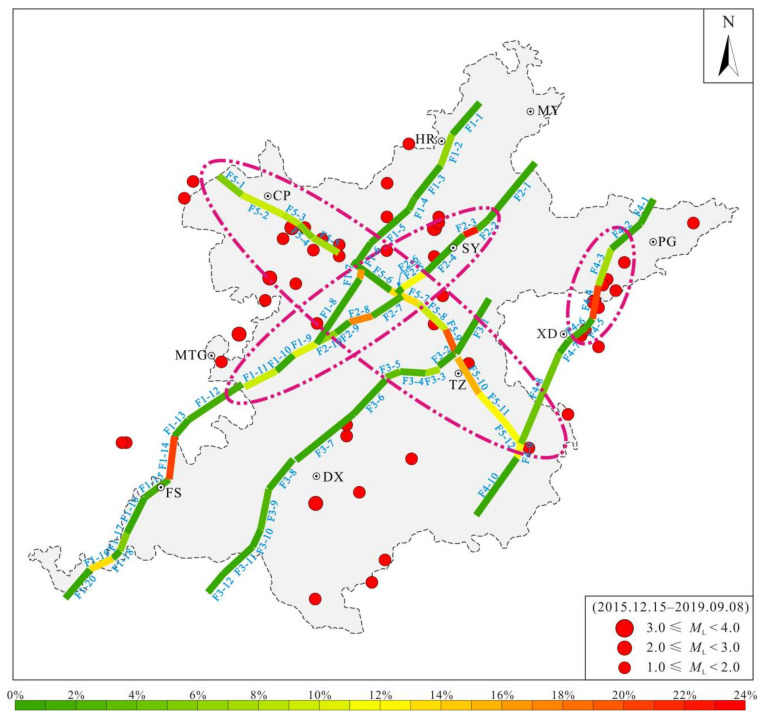
The initial fault slip potential (FSP) of the main concealed faults in the Beijing Plain.

Using the data discussed in Section 4.4.1, we analyzed the fault slip probability (FSP) of the major faults in the Beijing Plain in September 2019 (Figure 11). The calculations revealed that for the present in situ stress field, the slip probability of most of the faults in the Beijing Plain is less than 5%. Subsection F2-3 of the Shunyi fault has the highest slip probability (22%), followed by subsection F1-14 of the Huangzhuang-Gaoliying fault, subsection F2-8 of the Shunyi fault, subsection F4-4 of the Xiadian fault, and subsection F5-9 of the Nankou-Sunhe fault, which have probabilities of 17–20%. The subsections with slip probabilities of greater than 5% were mainly concentrated in the middle section of the Huangzhuang-Gaoliying fault (F1-7–F1-11), the middle and northern sections of the Shunyi fault (F2-3–F2-10), the northern section of the Xiadian fault (FF4-3–F4-4), and the northwestern (F5-1–F5-5) and southeastern sections (F5-7–F5-12) of the Nankou-Sunhe fault. The earthquake distribution was in suitable agreement with the calculated fault slip probability. In summary, under the current in situ stress field, the areas of the Beijing Plain with strong fault activity were mainly concentrated in the central and southeastern parts, mainly including the Nankou-Sunhe fault, the northern section of the Xiadian fault, and the areas where the main active faults intersect. The results provide scientific support for ensuring the urban geological security of the capital region. At the same time, the stability of building foundations is also a key aspect of urban construction [73,74,75,76], and the results also provide data support for this aspect.

## 5. Discussion

The formation and development of geological disasters such as ground fissures and land subsidence in the Beijing Plain are closely related to the fault activity. The monitoring results of ground deformation in the Beijing Plain from 2011 to 2013 revealed that the land subsidence area was mainly distributed along the Nankou-Sunhe fault and was concentrated in the central and southeastern parts of the plain. Before the 3.11 earthquake, under the influence of the NEE-trending principal stress field, the activity on the NE-trending normal faults in the region was relatively weaker than that on the NW-trending faults. After the 3.11 earthquake, the *S_H_* orientation changed from NEE to NE, and the activity on the NE-trending normal fault increased. The distributions of earthquakes in the Beijing Plain before and after the 3.11 earthquake also reflect the transition in the fault activity. From January 2000 to March 2011, the earthquakes were mainly distributed along the Nankou-Sunhe fault in the NW direction (Figure 9a). Although some of the earthquakes that occurred from December 2015 to September 2019 were still distributed along the Nankou-Sunhe fault, there was an obvious distribution trend along the NE direction, indicating that the activity on the NE-trending faults had increased. Since 2011, the ground at an airport in Beijing near the Shunyi fault has been damaged along the fault, and the vertical displacement of the ground fissures has reached 1–30 cm, which also shows that the activity of the Shunyi fault increased after the 3.11 earthquake. In summary, it can be inferred that the impact of the NE-trending faults in the Beijing Plain on geological disasters such as ground fissures and land subsidence increased after the 3.11 earthquake.

Numerous research results based on focal mechanism solutions, numerical simulations, and GPS observations have confirmed that the 3.11 earthquake resulted in the dynamic adjustment of the tectonic environment in eastern China. However, scientific issues such as the adjustment process of the tectonic environment in eastern China after the 3.11 earthquake and whether it has returned to the pre-earthquake state still require clarification. Hao, et al. (2020) [30] used observation data from continuous and mobile GPS stations from 1999 to 2017 to calculate the far-field coseismic and post-earthquake deformation displacement fields, and their results showed that the area affected by far-field coseismic deformation was obviously smaller than that affected by post-earthquake deformation. These areas mainly included the southeastern part of Northeast China and the Shandong Peninsula, and the relaxation time after the 3.11 earthquake was 1–11 years. Zhu (2020) [77] analyzed the crustal strain in the Bohai Rim region before and after the 3.11 earthquake based on GPS observation data and found that the 3.11 earthquake had a significant impact on the crustal deformation in the Bohai Rim region within three years after the earthquake. This region began to recover to the pre-earthquake state after 2014, but it has not yet fully recovered to the pre-earthquake state. The above studies all discussed the post-earthquake adjustment of the tectonic environment in eastern China from the perspective of GPS observations. Combined with the conclusions obtained from the analysis of in situ stress monitoring data in this study, it can be concluded that the tectonic environment in eastern China changed significantly after the 3.11 earthquake. The adjustment speed of the tectonic environment in the inland area of eastern China was faster than that near the subduction zone. The inland area began to recover back to the pre-earthquake tectonic environment after maintaining the nearly EW-stretching tectonic environment for about one year; while the area near the subduction zone, such as the Bohai Rim area, retained the nearly EW-stretching tectonic environment for about 3 years and then began to adjust back to the pre-earthquake tectonic environment. Currently, the tectonic environment in eastern China has not recovered to the pre-earthquake level.

**Figure 12:**
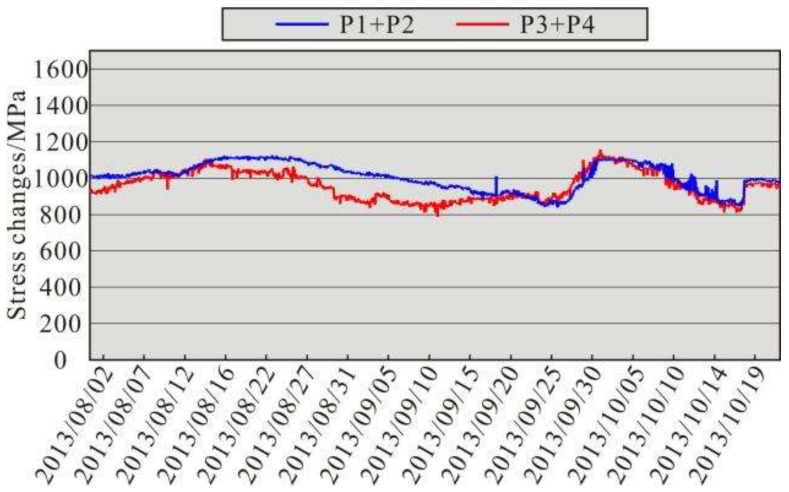
Self-verification curves of the stress monitoring data from the Changli piezomagnetic stress monitoring station.

This study was mainly based on real-time monitoring data from the Changli piezomagnetic stress monitoring station at a depth of 100.0 m. Therefore, the reliability of the in situ stress monitoring data directly affects the analysis of the in situ stress field and fault stability. The determination of the plane stress state requires three independent components. By measuring the respective deformations of the vertical borehole wall in three independent directions and based on the law of elastic deformation of a circular hole in a uniform stress field, the stress state in the horizontal plane can be calculated [78]. The Changli piezomagnetic stress monitoring station uses a four-component piezomangnetic stress gauge, which can simultaneously obtain the normal stress variations in four monitoring directions with an included angle of 45°. The four monitoring directions can be divided into two groups that are perpendicular to each other. Assuming that the wall of the borehole is an ideal elastic body, according to the influence of the partial stress tensor on the deformation of the circular hole, the sum of the changes in the monitoring values in the two measurement directions can be equal to conduct self-verification of the monitoring data [78]. Taking Changli Station as an example, that is, P1 + P2 = P3 + P4. However, in reality, crustal rocks have extensive vertical and lateral heterogeneity and are filled with fractures and pore fluids, which makes it difficult to ensure that the cross-section of the borehole is a regular circle. Factors such as changes in the geomagnetic field and the error of the instrument would have an impact on the monitoring results. Therefore, when analyzing piezomagnetic geostress monitoring data, it is difficult to verify the reliability of the monitoring data according to the ideal conditions, and the reliability of the data is usually judged from the trend of the two groups of data (P1 + P2) and (P3 + P4) [38,49]. Figure 12 shows the self-verification results of the monitoring curve for the Changli piezomagnetic stress monitoring station from August to October 2013. The change curves of the two groups of data (P1 + P2) and (P3 + P4) have similar shapes. Although they are not equal, the difference is small. Therefore, the positive stress changes monitored at the Changli monitoring station are relatively reliable. To avoid errors, the monitoring data were computed using the least-squares method.

There are some issues that still remain in this paper. We only discussed the stability of the five major Late Pleistocene–Holocene active faults in the Beijing Plain, and we did not consider the other faults. Moreover, we did not consider the difference in the gravitational field in the Beijing Plain when we analyzed the in situ stress field and fracture stability. It can be seen from Figure 7 that many strong earthquakes greater than magnitude 6 occurred near Japan after the 3.11 earthquake. However, by analyzing the monitoring data, we found that the monitoring data did not respond well to these strong earthquakes. The monitoring instrument did not monitor the changes in in situ stress before and after these strong earthquakes, so we could not analyze the impact of the strong earthquakes near Japan after the 3.11 earthquake on the in situ stress field and faults stability in the Beijing Plain. Limited by the placement depth of the monitoring instrument, the Changli Station can only obtain monitoring data at a depth of 100.0 m, which is far less than the buried depth of the faults in the Beijing Plain. Although the in situ stress data at the buried depth of the faults cannot be obtained, we assumed that the fault plane extends to the monitoring depth and combined the monitoring data and the Mohr–Coulomb failure criterion to analyze the faults activity. The analysis results still play a role in judging the activity of the deep fault. This has been proven feasible in previous studies [32,38,79]. In a follow-up study, we will make full use of the existing seismic geological data to deeply analyze the fault activity, seismic risk, and crustal stability in the Beijing Plain. In addition, to address the problem that the monitoring data are affected by factors such as the groundwater level, water temperature, changes in the geomagnetic field, and the error of the instrument itself, in the next stage, we will continue to promote in situ stress monitoring in the capital area and will strengthen the research and development of monitoring equipment to improve the monitoring precision and monitoring depth.

## 6. Conclusions

Based on the in situ stress monitoring data from Changli Station, in this study, the dynamic adjustment of the in situ stress field after the 3.11 earthquake and the current characteristics of the in situ stress field in the Beijing Plain area were investigated, and the stability of five main active faults in the plain area was assessed. The main conclusions of this study are as follows.

(1) After the 3.11 earthquake in Japan, the in situ stress field in the Beijing Plain changed from nearly EW extrusion to nearly EW extension. This state was maintained until June 2012, after which it began to gradually adjust back to the pre-earthquake state. In September 2019, at a depth of 100.0 m in the Beijing Plain, *S_H_* was 5.30–5.62 MPa, *S_h_* was 3.61–3.93 MPa, and the *S_H_* orientation was N 39.0–39.6° E. The tectonic environment of the Beijing Plain had not yet recovered to the pre-earthquake state.

(2) The results of the quantitative analysis of the fault stability revealed that before the 3.11 earthquake, under the action of the NNE-trending principal compressive stress, the *μ* values of the subsections of the five main faults in the Beijing Plain were 0.1–0.34, and the stress accumulation was at a low to medium level. The Huangzhuang-Gaoliying fault, the Xiadian fault, and the Nankou-Sunhe fault had the strongest activity, followed by Shunyi fault, and the Nanyuan-Tongxian fault had the weakest activity. Bounded by the Nankou-Sunhe fault, the fault activity was greater in the northeastern region of the Beijing Plain than in the southwestern region.

From the 3.11 earthquake to June 2012, affected by the adjustment of the direction of the principal compressive stress field from NEE to NNE, the *μ* values of the subsections of the five main faults were 0.28–0.52, and the stress accumulation level increased. The activity on the Shunyi fault, the Nanyuan-Tongxian fault, and the southern and central sections of the Huangzhuang-Gaoliying fault increased significantly, while the activity on the northern section of the Huangzhuang-Gaoliying fault and the Xiadian fault decreased, except for individual subsections. The activity on the northwestern and middle sections of the Nankou-Sunhe fault weakened, and the activity on the southeastern section increased.

From June 2012 to September 2019, under the action of the NE-trending principal compressive stress field, the *μ* values of the subsections of the five main faults in the Beijing Plain were 0.06–0.29, and the stress accumulation level was lower than that before the 3.11 earthquake. The activity on the southern and central sections of the Huangzhuang-Gaoliying fault, the Xiadian fault, and the northwestern and southeastern sections of the Nankou-Sunhe fault was relatively strong.

(3) According to the results of the quantitative analysis of the fault stability, in each stage of the adjustment of the in situ stress field in the Beijing Plain, the calculated *μ* values of the subsections of the five main faults did not exceed 0.6, and the critical friction coefficients of the main concealed faults in the Beijing Plain were 0.22–0.52.

(4) The fault slip potential analysis results revealed that under the current in situ stress environment, most of the fault subsections in the Beijing Plain had a low slip potential (FSP < 5%). The fault subsection with the highest slip potential was subsection F2-3 of the Shunyi fault (22%), followed subsection F1-14 of the Huangzhuang-Gaoliying fault, subsection F2-8 of the Shunyi fault, subsection F4-4 of the Xiadian fault, and subsection F5-9 of the Nankou-Sunhe fault, which had FSP values of 17–20%. The areas with high fault slip potential were mainly concentrated in the central and southeastern parts of the Beijing Plain, including the Nankou-Sunhe fault, the northern section of the Xiadian fault, and the areas where the five main active faults intersect.

## Figures and Tables

**Table 1:** Dynamic adjustment of stress field of the Beijing Plain after the Tohoku-Oki 3.11 M9.0 earthquake.

Date (Time)	Component Variation (MPa)				*S_H_* (MPa)	*S_h_* (MPa)	*S_H_* Orientation
	∆σ_1_	∆σ_2_	∆σ_3_	∆σ_4_			
11 March 2011	0	0	0	0	6.04	4.35	N 77–88° E
6 June 2012 (00:00)	−0.627	−1.229	−2.465	1.671	6.04–6.35	2.71–3.02	N 26.9–28.7° E
4 May 2013 (05:00)	−1.317	−1.761	−2.408	−0.599	4.17–4.49	2.86–3.17	N 42.9–46.4° E
16 June 2015 (02:00)	−1.599	−1.718	−1.557	0.835	5.00–5.32	3.04–3.36	N 40.8–42.1° E
15 December 2015 (10:00)	−1.570	−1.848	−1.404	1.998	5.70–6.01	2.96–3.28	N 32.4–33.5° E
8 September 2019 (18:00)	−1.755	−2.144	−0.339	1.925	5.30–5.62	3.61–3.93	N 39.0–39.6° E

**Table 2:** Characteristic parameters of the subsections of the main concealed faults in the Beijing Plain.

Fault	Subsection	Strike	Dip Angle	Length (km)
Huangzhuang-Gaoliying Fault (F1)	F1-1	41.0° ± 5°	63° ± 5°	8.95
	F1-2	20.2° ± 5°	63° ± 5°	7.00
	F1-3	38.5° ± 5°	63° ± 5°	8.94
	F1-4	26.4° ± 5°	63° ± 5°	2.7
	F1-5	48.8° ± 5°	63° ± 5°	10.77
	F1-6	34.2° ± 5°	63° ± 5°	5.52
	F1-7	10.9° ± 5°	67° ± 5°	2.03
	F1-8	33.7° ± 5°	67° ± 5°	16.52
	F1-9	63.6° ± 5°	67° ± 5°	5.47
	F1-10	47.2° ± 5°	67° ± 5°	4.91
	F1-11	63.0° ± 5°	67° ± 5°	7.53
	F1-12	56.4° ± 5°	67° ± 5°	13.71
	F1-13	40.6° ± 5°	67° ± 5°	4.60
	F1-14	6.9° ± 5°	67° ± 5°	9.20
	F1-15	53.3° ± 5°	65° ± 5°	6.49
	F1-16	26.6° ± 5°	65° ± 5°	8.42
	F1-17	20.9° ± 5°	65° ± 5°	4.03
	F1-18	43.0° ± 5°	65° ± 5°	2.27
	F1-19	64.6° ± 5°	65° ± 5°	5.37
	F1-20	40.5° ± 5°	65° ± 5°	8.04
Shunyi fault (F2)	F2-1	39.5° ± 5°	70° ± 5°	15.67
	F2-2	48.7° ± 5°	60° ± 5°	3.06
	F2-3	66.2° ± 5°	60° ± 5°	3.11
	F2-4	45.6° ± 5°	60° ± 5°	11.75
	F2-5	57.3° ± 5°	70° ± 5°	5.53
	F2-6	45.0° ± 5°	70° ± 5°	1.68
	F2-7	56.5° ± 5°	70° ± 5°	7.63
	F2-8	75.1° ± 5°	70° ± 5°	4.98
	F2-9	51.3° ± 5°	70° ± 5°	3.62
	F2-10	63.3° ± 5°	70° ± 5°	4.34
Nanyuan-Tongxian fault (F3)	F3-1	31.1° ± 5°	80° ± 5°	13.58
	F3-2	51.6° ± 5°	80° ± 5°	4.31
	F3-3	74.8° ± 5°	80° ± 5°	3.12
	F3-4	273.7° ± 5°	80° ± 5°	5.39
	F3-5	68.0° ± 5°	80° ± 5°	3.25
	F3-6	43.3° ± 5°	80° ± 5°	10.11
	F3-7	51.2° ± 5°	80° ± 5°	15.63
	F3-8	39.2° ± 5°	80° ± 5°	8.18
	F3-9	11.4° ± 5°	80° ± 5°	8.78
	F3-10	24.7° ± 5°	80° ± 5°	4.02
	F3-11	47.4° ± 5°	80° ± 5°	8.36
	F3-12	39.9° ± 5°	80° ± 5°	5.26
Xiadian fault (F4)	F4-1	27.8° ± 5°	65° ± 5°	5.92
	F4-2	49.6° ± 5°	65° ± 5°	8.16
	F4-3	19.4° ± 5°	65° ± 5°	8.34
	F4-4	9.9° ± 5°	65° ± 5°	7.13
	F4-5	35.5° ± 5°	65° ± 5°	2.20
	F4-6	52.8° ± 5°	65° ± 5°	4.84
	F4-7	36.6° ± 5°	65° ± 5°	3.12
	F4-8	23.0° ± 5°	65° ± 5°	20.94
	F4-9	16.3° ± 5°	65° ± 5°	3.16
	F4-10	35.3° ± 5°	65° ± 5°	14.94
Nankou-Sunhe fault (F5)	F5-1	308.8° ± 5°	68° ± 5°	6.45
	F5-2	295.6° ± 5°	68° ± 5°	9.78
	F5-3	300.8° ± 5°	68° ± 5°	3.87
	F5-4	315.1° ± 5°	68° ± 5°	4.03
	F5-5	300.4° ± 5°	68° ± 5°	6.45
	F5-6	306.8° ± 5°	74° ± 5°	10.20
	F5-7	295.2° ± 5°	66° ± 5°	6.36
	F5-8	313.1° ± 5°	66° ± 5°	8.12
	F5-9	336.6° ± 5°	66° ± 5°	5.64
	F5-10	328.9° ± 5°	66° ± 5°	8.91
	F5-11	317.8° ± 5°	66° ± 5°	8.24
	F5-12	323.6° ± 5°	66° ± 5°	5.51

**Table 3:** Parameters error of the analysis of the slip potential on the main active faults.

Parameter (Units)	Mean Value	Error Value
*S_H_* (MPa)	5.46	±0.16
*S_h_* (MPa)	3.77	±0.16
*S_H_* orientation (°)	39.3	±0.3
Faults strike (°)	As shown in Table 2	±5
Faults dip (°)	As shown in Table 2	±5
Pore water pressure (MPa)	0.75	±0.05
Critical fault coefficient	0.37	±0.15

## Data Availability

All data can be obtained from the corresponding author by request.
